# A deep learning quantified stroma-immune score to predict survival of patients with stage II–III colorectal cancer

**DOI:** 10.1186/s12935-021-02297-w

**Published:** 2021-10-30

**Authors:** Zeyan Xu, Yong Li, Yingyi Wang, Shenyan Zhang, Yanqi Huang, Su Yao, Chu Han, Xipeng Pan, Zhenwei Shi, Yun Mao, Yao Xu, Xiaomei Huang, Huan Lin, Xin Chen, Changhong Liang, Zhenhui Li, Ke Zhao, Qingling Zhang, Zaiyi Liu

**Affiliations:** 1 Department of Radiology, Guangdong Provincial People’s Hospital, Guangdong Academy of Medical Sciences, 106 Zhongshan Er Road, Guangzhou, 510080 China; 2 School of Medicine, South China University of Technology, Panyu District, Guangzhou, 510006 China; 3 Department of General Surgery, Guangdong Provincial People’s Hospital, Guangdong Academy of Medical Sciences, Guangzhou, 510080 China; 4 Department of Radiology, Zhuhai People’s Hospital, Zhuhai Hospital Affiliated with Jinan University, Zhuhai, 519000 China; 5 Department of Pathology, The Sixth Affiliated Hospital of Sun Yat-Sen University, Guangzhou, 510655 China; 6 Department of Pathology, Guangdong Provincial People’s Hospital, Guangdong Academy of Medical Sciences, Guangzhou, 510080 China; 7 Department of Radiology, The First Affiliated Hospital of Chongqing Medical University, Chongqing, 400016 China; 8 School of Bioengineering, Chongqing University, Chongqing, 400044 China; 9 The Second School of Clinical Medicine, Southern Medical University, Guangzhou, 510080 China; 10 Department of Radiology, Guangzhou First People’s Hospital, Guangzhou, 510180 China

**Keywords:** Deep learning, Whole-slide images, Tumor-infiltrating lymphocytes, Colorectal cancer

## Abstract

**Background:**

Profound heterogeneity in prognosis has been observed in colorectal cancer (CRC) patients with intermediate levels of disease (stage II–III), advocating the identification of valuable biomarkers that could improve the prognostic stratification. This study aims to develop a deep learning-based pipeline for fully automatic quantification of immune infiltration within the stroma region on immunohistochemical (IHC) whole-slide images (WSIs) and further analyze its prognostic value in CRC.

**Methods:**

Patients from two independent cohorts were divided into three groups: the development group (N = 200), the internal (N = 134), and the external validation group (N = 90). We trained a convolutional neural network for tissue classification of CD3 and CD8 stained WSIs. A scoring system, named stroma-immune score, was established by quantifying the density of CD3^+^ and CD8^+^ T-cells infiltration in the stroma region.

**Results:**

Patients with higher stroma-immune scores had much longer survival. In the development group, 5-year survival rates of the low and high scores were 55.7% and 80.8% (hazard ratio [HR] for high vs. low 0.39, 95% confidence interval [CI] 0.24–0.63, P < 0.001). These results were confirmed in the internal and external validation groups with 5-year survival rates of low and high scores were 57.1% and 78.8%, 63.9% and 88.9%, respectively (internal: HR for high vs. low 0.49, 95% CI 0.28–0.88, P = 0.017; external: HR for high vs. low 0.35, 95% CI 0.15–0.83, P = 0.018). The combination of stroma-immune score and tumor-node-metastasis (TNM) stage showed better discrimination ability for survival prediction than using the TNM stage alone.

**Conclusions:**

We proposed a stroma-immune score via a deep learning-based pipeline to quantify CD3^+^ and CD8^+^ T-cells densities within the stroma region on WSIs of CRC and further predict survival.

**Supplementary Information:**

The online version contains supplementary material available at 10.1186/s12935-021-02297-w.

## Introduction

Colorectal cancer (CRC) is one of the leading causes of cancer-associated death worldwide [1]. Currently, therapeutic decisions and prognostic evaluations of CRC are mainly performed by the tumor-node-metastasis (TNM) staging system [2]. However, the TNM staging system fails to provide complete prognostic information as diverse prognoses are observed among patients with the same stage due to the differences in clinical and molecular phenotypes, patterns of genetic damage, and host immune responses [3, 4]. In particular, although the treatment strategies such as surgery, chemotherapy, radiotherapy, and immunotherapy have improved obviously, profound heterogeneity in prognosis has been observed in CRC patients with intermediate levels of disease (stage II–III) [4]. Hence, biomarkers that could improve the prognostic stratification for patients with stage II–III CRC are urgently needed.

The increased knowledge of the immune system’s central role in tumor progression advocates identifying prognostic biomarkers to describe immune infiltration. In particular, the Immunoscore, which is obtained from the densities of CD3^+^ and CD8^+^ T-cells in the tumor center and invasive margin, has been reported to hold superior and independent prognostic value over the traditional TNM system in patients with stage II–III CRC [5, 6]. Recently, a more detailed immune infiltration analysis approach has been proposed to describe the quantitative landscape of tumor-immune microenvironment (TIME) in CRC by quantifying tumor-infiltrating lymphocytes (TILs) in the stromal and intraepithelial regions, respectively, with inspiring results [7]. This advocate further interest in the tumor immune cell infiltration in one of the major constituents of the TIME, the stroma. Additionally, as shown by the study of Kather et al. [8], the information from non-tumor components (such as stroma) could provide more prognostic value than tumor epithelium. The tumor stroma characteristics, such as the tumor-stroma ratio (TSR), have been well supported by emerging studies as an independent prognostic tool in CRC [9, 10].

With the recent advance of artificial intelligence technologies and digital whole-slide images (WSIs), it is possible to identify novel biomarkers from automatically segmented histological components [11]. Previous studies conducted automatic quantification of TILs based on multiplex immunofluorescence WSIs, suggesting that TILs combined with other risk factors can improve the accuracy of prognosis prediction in CRC patients [12, 13]. Therefore, inspired by previous observations [5, 7, 10], we postulate that describing the immune infiltration lymphocytes (CD3^+^ and CD8^+^ T-cells) in the stroma region using a deep learning approach could further refine the prognostic stratification of patients with stage II–III CRC.

Therefore, the goal of this study is to propose a deep learning-based pipeline for fully automated quantification of immune infiltration within the stroma region on the immunohistochemical (IHC) WSIs and further analyze its prognostic value in patients with CRC.

## Methods

### Patients and follow up

This retrospective study was approved by the Research Ethics Committee of Guangdong Provincial People's Hospital (Cohort 1) and the Sixth Affiliated Hospital of Sun Yat-sen University (Cohort 2), and the informed consent was waived. The institutional medical record database was analyzed to identify patients with histologically confirmed stage II–III CRC patients who underwent surgical resection with curative intent from Mar 2009 to Dec 2014 at Cohort 1 and Jan 2013 to Dec 2014 at Cohort 2. Patients with follow-up information and IHC (CD3 and CD8) WSIs available were included in the study. Patients who received neoadjuvant therapy or died within 30 days after surgery were excluded. Moreover, patients with incomplete clinical information and poor image quality were also excluded. After enrollment, patients from Cohort 1 were randomly divided into two groups: 60% of patients formed the development group, whereas 40% formed the internal validation group. Patients from Cohort 2 formed the external validation group.

Clinicopathological factors were collected from medical records, including age, sex, tumor site (colon/rectum), T-category, N-category, TNM stage, microsatellite instability (MSI) status, and treatment modalities. MSI status was determined by IHC with the expression of mismatch repair proteins (MLH1, MSH2, MSH6, and PMS2) and classified as MSI and microsatellite stable (MSS). The outcome of interest was overall survival (OS). The follow-up methods included clinical consultations, medical records reviews, and telephone interviews.

### Datasets for tissue classification

The CD3 and CD8 IHC stained tissue sections were imaged using digital Whole Slide Scanning (Aperio-AT2, Leica, USA) at 40× magnification. CRC tissues were grouped into nine types: tumor epithelium, tumor stroma, adipose, background, debris, lymphocytes, mucus, smooth muscle, and normal mucosa. For decomposing different tissue types on IHC WSIs, we used two tiles datasets to train a tissue classification model and one tile dataset to test the model. An open available hematoxylin and eosin (HE) tiles dataset consisting of 283 k tissue tiles was used as the HE tiles dataset [8, 10]. An IHC tiles training dataset consisting of 154.4 k tissue tiles was established from 242 CD3 and CD8 slides of 121 patients in the development group. An IHC tiles test dataset consisting of 22.5 k tissue tiles was also established from 114 slides of 57 patients in the internal validation group. Details of the IHC staining and datasets generation are presented in Additional file 1.

### Tissue segmentation on IHC WSIs

**Fig. 1:**
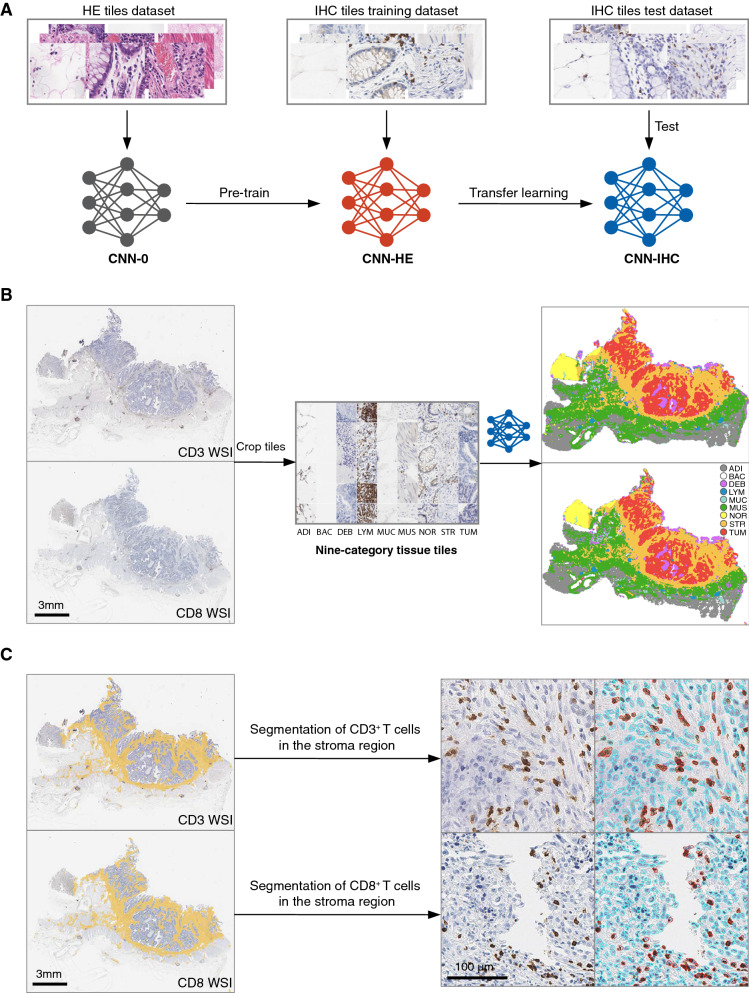
Study workflow. **A** Convolutional neural networks for colorectal tissue classification. First, a HE tiles dataset was used to pre-train an untrained VGG-19 network (CNN-0) as the CNN-HE model. Next, an IHC tiles training dataset was used to train the CNN-HE model as the CNN-IHC model with transfer learning. An independent dataset was used to test the tissue tiles classification performance of the CNN-IHC model. **B** Rough segmentation of IHC (CD3 and CD8) WSI. The CNN-IHC model was used to perform patch-level segmentation of IHC WSI. **C** Immune cells in the stroma region. The segmented stroma region was mapped on the original WSI, and CD3^+^ and CD8^+^ T-cells in this region were segmented and counted. *HE* hematoxylin–eosin, *IHC* immunohistochemical, *WSI* whole-slide image, *ADI* adipose, *BAC* background, *DEB* debris, *LYM* lymphocytes, *MUC* mucus, *MUS* muscle, *NOR* normal mucosa, *STR* stroma, *TUM* tumor epithelium

A convolutional neural network (CNN) was trained for tissue classification of CD3 and CD8 stained WSIs. First, we used the HE tiles dataset to train a VGG-19 model (CNN-0) with random initialization. Then we fine-tuned the trained model (CNN-HE) by transfer learning with the IHC tiles training dataset to generate a CNN-IHC model. Finally, the CNN-IHC model’s classification performance was evaluated using the IHC tiles test dataset (Fig. 1A). Specifically, at the pre-train stage, the HE tiles dataset (283 k) was used to train a VGG-19 model for HE tiles nine categories classification. 10% of the samples were randomly selected as an internal validation set to monitor the training process. The training procedure generally followed Simonyan et al. [14], except for setting the batch size to 64. At the transfer learning stage, the IHC tiles training dataset (154.4 k tiles) served as the training set. Fine-tuning was used to train the CNN-HE model with SGDM. The mini-batch size was set as 64, and a fixed learning rate of 3 × 10^−4^ was used to train the model for ten epochs, generating a CNN-IHC model. We trained the network on a desktop workstation with one NVIDIA GeForce RTX 2080Ti GPU. The CNN-IHC model training and testing were done using in MATLAB environment (R2020a, MathWorks, USA). The trained CNN-IHC model is available online (https://doi.org/10.5281/zenodo.5589269).

In the rough segmentation step, the CD3 and CD8 stained WSIs were scaled to 20× magnification. Then overlapped tiles (224 pixels × 224 pixels) were extracted from WSIs with a 75-pixel overlapped border. The CNN-IHC model classified the cropped tile as one tissue type with the maximum probability (Fig. 1B).

### Stroma-immune score

The stroma region segmentation result was mapping on the CD3 and CD8 WSIs as the region of interest (ROI). Then the positive CD3/CD8 T-cells (CD3^+^/CD8^+^) that presented as the brown color within the ROI were segmented and counted by an in-house program. The mean density of positive cells in the stroma region was calculated (Fig. 1C). The immune cell density of each patient was converted as the percentile value (range from 0 to 100%) according to the immune cell distribution in the development group.

Hence, CD3 and CD8 percentiles were obtained, and the average of these two percentiles was calculated to obtain a stroma-immune score (percentile). According to the stroma-immune score (percentile) distribution in the development group, patients were divided into three categories (high, intermediate, and low) to obtain the three-category stroma-immune score. These two thresholds were determined in the development group to balance the proportion of patients in each category by using the cut2 function from the *Hmisc* R package [15]. Then the intermediate and high scores were combined into a new high score, forming a two-category score (low vs. high). All analysis steps would be tested in the internal and external validation groups.

### Evaluation of the stroma-immune score

For the three- or two-category stroma-immune score, the Kaplan–Meier method was used to analyze the survival curves. The log-rank test was used to test the differences in survival distributions. The Cox proportional hazards model was used to compute the hazard ratio (HR) of the stroma-immune score and other clinicopathological risk factors (age, sex, TNM stage, and tumor site) for OS. Subgroup analyses were performed by age, sex, TNM stage, tumor site, MSI status, and treatment modalities in Cohort 1. The performance of stroma-immune score and other factors were assessed by Harrell’s C-index with 1000 times bootstrap.

### Stroma-immune score and TSR

We also calculated the TSR, which was defined as the proportion of stroma area in the sum area of tumor epithelium and stroma in the WSI. Patients were grouped as stroma-low and stroma-high using a fixed threshold of 50%. In stroma-low and stroma-high subgroups, Kaplan–Meier curves were plotted for the three categories stroma-immune score in Cohort 1.

### Stroma-immune score and intraepithelial-immune score

We further established an intraepithelial-immune score, which summarized the mean density of CD3^+^ and CD8^+^ T-cells immune infiltration in the intraepithelial tumor. The intraepithelial-immune score was calculated the same way as the stroma-immune score, except that the ROI was replaced by the tumor epithelium region in Cohort 1. The Pearson correlation coefficient [16] of the stroma-immune score and intraepithelial-immune score was calculated, and multivariate analysis was also performed.

### Statistical analysis

All statistical analyses were performed with R software (version 3.6.1) [17]. Clinicopathological characteristics were compared among the three groups by Kruskal–Wallis rank sum test or Chi-square test when appropriate. Multiple comparisons correction was applied by Bonferroni correction. Two-sided P < 0.05 was considered statistically significant.

## Results

### Patients

A total of 424 patients were included in our study (Additional file 1: Fig. S1). The development group included 200 patients (aged 63.55 ± 11.21 years), the internal validation group included 134 patients (62.78 ± 13.31 years), and the external validation group included 90 patients (62.34 ± 12.74 years). In Cohort 1, 147 (44.0%) patients had stage II colorectal cancer and 187 (56.0%) patients had stage III colorectal cancer. In Cohort 2, there were 45 (50.0%) and 45 (50.0%) patients with stage II and III rectal cancer, respectively. The median follow-up time was 76 months in Cohort 1 and 69.5 months in Cohort 2. The 5-year survival rate was 71.5% (95% CI 66.9–76.5%) in Cohort 1 and 82.0% (74.4–90.4%) in Cohort 2. There were no statistical significances between the three groups in terms of age, sex, T-category, N-category, and TNM stage (all P > 0.05; Table 1), except for the tumor site. Perhaps because of the lack of colon cancer patients in the external validation group.

**Table 1:** The distributions of demographic and clinicopathologic characteristics of colorectal cancer patients in the three groups

	Development group	Internal validation group	External validation group	P
Age (years)				0.845^#^
Mean	63.55	62.78	62.34	
SD	11.21	13.31	12.74	
Sex				0.134^##^
Male	129 (64.5%)	62 (53.7%)	56 (62.2%)	
Female	71 (35.5%)	72 (46.3%)	34 (37.8%)	
T-category				0.161^##^
T1	1 (0.5%)	0 (0%)	2 (2.2%)	
T2	10 (5.0%)	4 (3.0%)	3 (3.3%)	
T3	168 (84.0%)	119 (88.8%)	82 (91.1%)	
T4	21 (10.5%)	11 (8.2%)	3 (3.3%)	
N-category				0.280^##^
N0	82 (41.0%)	64 (47.8%)	45 (50%)	
N1	71 (35.5%)	46 (34.3%)	33 (36.7%)	
N2	47 (23.5%)	24 (17.9%)	12 (13.3%)	
Stage				0.240^##^
II	82 (41.0%)	65 (48.5%)	45 (50%)	
III	118 (59.0%)	69 (51.5%)	45 (50%)	
Tumor site				< 0.001^##^
Colon	115 (57.5%)	73 (54.5%)	0 (0%)	
Rectum	85 (42.5%)	61 (45.6%)	90 (100%)	

^#^Kruskal–Wallis rank sum test

^##^Chi-square test

### Tissue classification performance

**Fig. 2:**
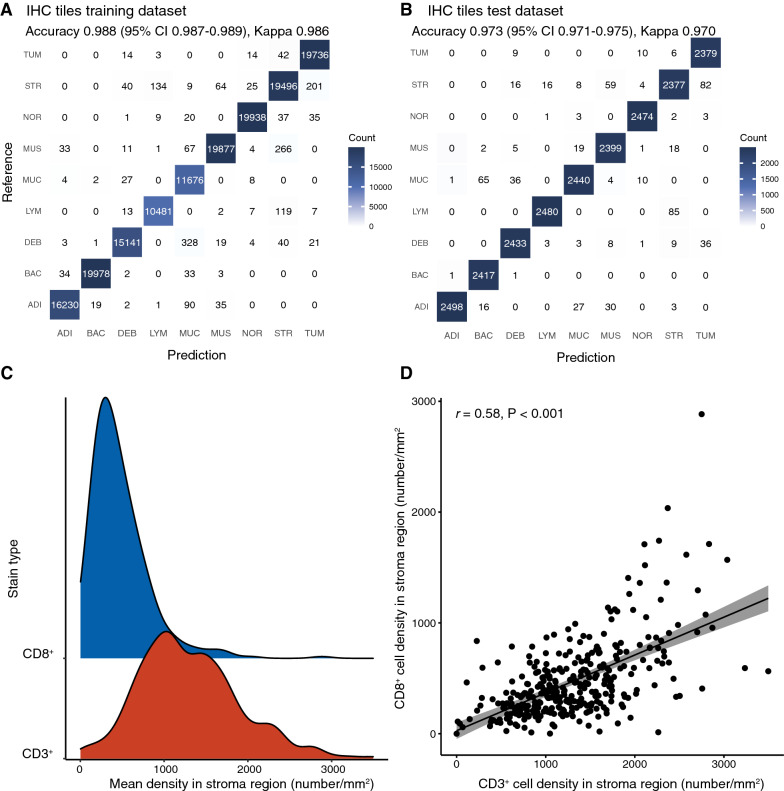
IHC tissue tiles classification performance on the IHC tiles training (**A**) and test (**B**) sets. **C** Mean density distributions of the two cell counting results. **D** Correlation analysis between the CD3^+^ mean density and the CD8^+^ mean density in the stroma region. *IHC* immunohistochemical

High classification accuracy performance was achieved in all tissue classes by CNN-IHC model (IHC tiles training dataset: 0.988, 95% confidence interval [CI] 0.987–0.989; IHC tiles test dataset: 0.973, 95% CI 0.971–0.975), which could be observed from the confusion matrixes (Fig. 2A, B).

### Prognostic value of the stroma-immune score

The CD3^+^ and CD8^+^ mean densities in the stroma region were presented in Fig. 2C. A moderate correlation was observed (r = 0.58, P < 0.001) between CD3^+^ and CD8^+^ T-cells mean densities (Fig. 2D). Patients were classified into low, intermediate, and high stroma-immune score groups based on 40% and 63.5% thresholds, splitting patients into three percentile groups in the development group.

**Fig. 3:**
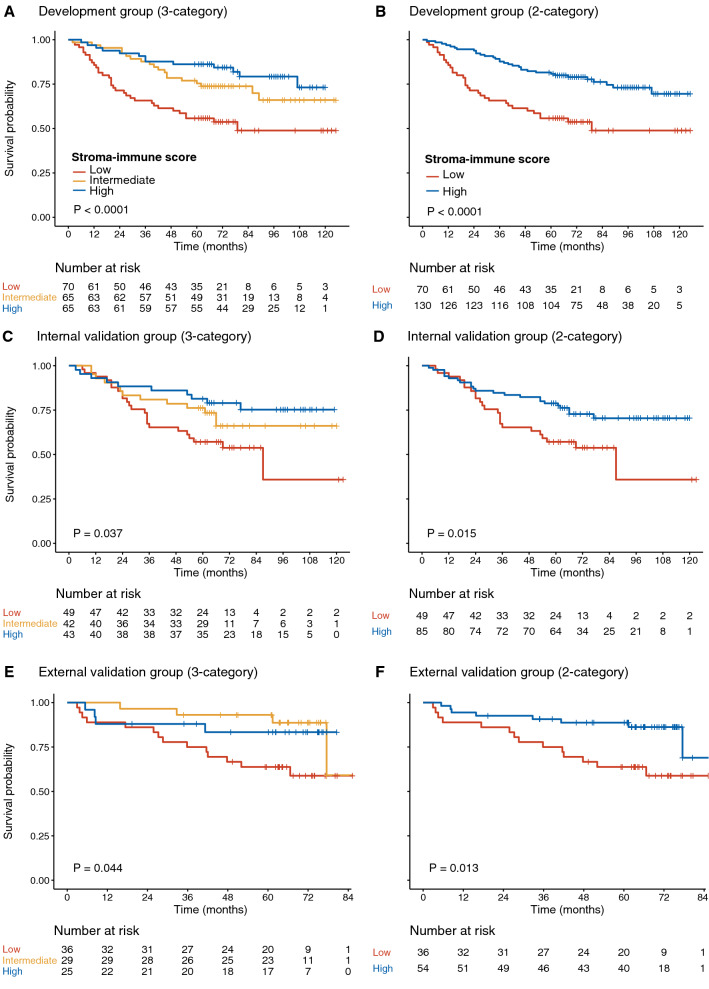
Kaplan–Meier curves analysis for the stroma-immune score in development, internal and external validation group. **A**, **C**, **E** Stroma-immune score (3-category); **B**, **D**, **F** Stroma-immune score (2-category, the intermediate and high score were combined into a new high score)

Patients with higher stroma-immune scores had much longer survival. Five-year survival rates of low, intermediate, and high stroma-immune score in the development group were 55.7%, 75.4%, and 86.2% (HR for high vs. low 0.30, 95% CI 0.16–0.58, P < 0.001; Fig. 3A; Table 2). These results were confirmed in the internal validation group: the survival rates at 5 years were 57.2% in the low group, 76.2% in the intermediate group, and 81.4% in the high group (HR for high vs. low 0.40, 95% CI 0.19–0.85, P = 0.017; Fig. 3C), while no significant difference was found in external validation group (HR for high vs. low 0.40, 95% CI 0.13–1.22, P = 0.110; Fig. 3E). When the stroma-immune score was classified into two categories, the intermediate and high scores were combined into a new high score, patients with the new high stroma-immune score still showed significant prolonged OS in the development group with 5-year survival rates of the low and high scores were 55.7% and 80.8% (HR for high vs. low 0.39, 95% CI 0.24–0.63, P < 0.001; Fig. 3B; Table 3), and the results were further confirmed in the internal validation group with 5-year survival rates of the low and high score were 57.1% and 78.8% (HR for high vs. low 0.49, 95% CI 0.28–0.88, P = 0.017; Fig. 3D), and external validation group with 5-year survival rates of the low and high score were 63.9% and 88.9% (HR for high vs. low 0.35, 95% CI 0.15–0.83, P = 0.018; Fig. 3F).

**Table 2:** Unadjusted and multivariate analyses for overall survival (3-category stroma-immune score)

	Development group			Internal validation group			External validation group		
	HR	95% CI	P	HR	95% CI	P	HR	95% CI	P
Unadjusted stratified Cox model									
Stroma-immune score									
Low	1			1			1		
Intermediate	0.48	0.27–0.84	0.011	0.60	0.30–1.18	0.140	0.31	0.10–0.94	0.039
High	0.30	0.16–0.58	< 0.001	0.40	0.19–0.85	0.017	0.40	0.13–1.22	0.110
Multivariable stratified Cox model									
Stroma-immune score									
Low	1			1			1		
Intermediate	0.45	0.25–0.79	0.006	0.60	0.30–1.19	0.15	0.34	0.11–1.06	0.062
High	0.35	0.18–0.66	0.001	0.43	0.20–0.92	0.029	0.40	0.13–1.23	0.110
TNM stage									
II	1			1			1		
III	3.25	1.76–6.00	< 0.001	2.63	1.40–4.95	0.003	1.77	0.75–4.20	0.195
Age	1.04	1.01–1.06	0.007	1.02	0.99–1.04	0.15	1.03	1.00–1.07	0.072

*HR* hazard ratio, *CI* confidence interval, *TNM* tumor-node-metastasis

By stratified analysis with TNM stage, age, sex, and tumor site, the stroma-immune score (3-category) remained a statistically significant predictor for OS at Cohort 1 (all P < 0.05; Additional file 1: Fig. S2). MSI status was available in 257 patients in Cohort 1, including 27 patients with MSI and 230 patients with MSS. When stroma-immune score stratified into two categories by combining intermediate and high groups into a new high group, patients with high stroma-immune score had prolonged OS in both MSI and MSS subgroup (MSI: unadjusted HR for high vs. low 0.11, 95% CI 0.01–1.03, P = 0.053; and MSS: 0.50, 0.32–0.77, 0.002; Additional file 1: Fig. S3). Treatment modalities were available in 130 patients in the whole cohort: 47 were treated with surgery alone, and 83 with surgery and adjuvant chemotherapy. There was no significant difference in the stroma-immune score (2-category) for OS in both treatment groups (P > 0.05; Additional file 1: Fig. S4).

In the multivariate analysis, the TNM stage, age, and stroma-immune score (2-category) were identified as independent predictors for OS, patients with a high stroma-immune score associated with better OS in the development group (HR for high vs. low 0.40, 95% CI 0.24–0.66, P < 0.001), internal validation group (HR for high vs. low 0.52, 95% CI 0.29–0.93, P = 0.027), and external validation group (HR for high vs. low 0.37, 95% CI 0.15–0.89, P = 0.027; Table 3). Additional file 1: Fig. S5 shows how the pipeline can be used to predict the prognosis of one patient with CD3 and CD8 WSIs.

**Table 3:** Unadjusted and multivariate analyses for overall survival (2-category stroma-immune score)

	Development group			Internal validation group			External validation group		
	HR	95% CI	P	HR	95% CI	P	HR	95% CI	P
Unadjusted stratified Cox model									
Stroma-immune score									
Low	1			1			1		
High	0.39	0.24–0.63	< 0.001	0.49	0.28–0.88	0.017	0.35	0.15–0.83	0.018
Multivariable stratified Cox model									
Stroma-immune score									
Low	1			1			1		
High	0.40	0.24–0.66	< 0.001	0.52	0.29–0.93	0.027	0.37	0.15–0.89	0.027
TNM stage									
II	1			1			1		
III	3.31	1.80–6.09	< 0.001	2.68	1.42–5.04	0.002	1.79	0.76–4.22	0.184
Age	1.04	1.01–1.06	0.006	1.02	0.99–1.04	0.151	1.03	1.00–1.07	0.072

*HR* hazard ratio, *CI* confidence interval, *TNM* tumor-node-metastasis

### Stroma-immune score and TSR

**Fig. 4:**
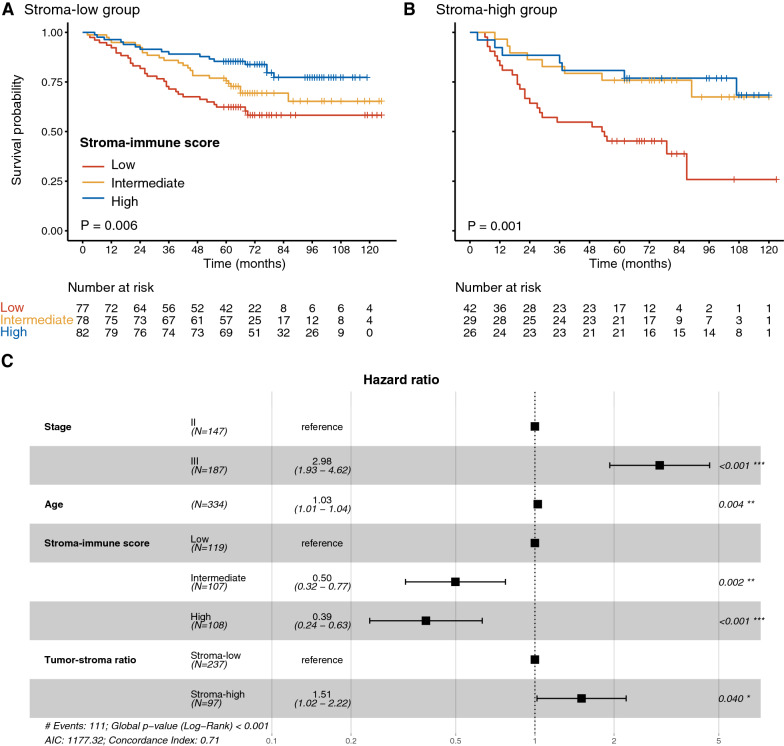
**A** Kaplan–Meier survival curves for the stroma-immune score in the stroma-low group. **B** Kaplan–Meier survival curves for the stroma-immune score in the stroma-high group. **C** HRs with 95% CI in the multivariable Cox model, including TNM stage (II–III), sex, age, stroma-immune score, and stroma-tumor ratio. *HR* hazard ratio, *CI* confidence interval

For Cohort 1, patients with stroma-high had worse OS (HR for stroma-high vs. stroma-low 1.48, 95% CI 1.01–2.19, P = 0.046; Additional file 1: Fig. S6). In the stroma-low group, the stroma-immune score still has a prognostic value, wherein patients with high stroma-immune scores had the best outcome (Fig. 4A). In the stroma-high group, patients with low stroma-immune scores had the worst survival (Fig. 4B). When we performed the multivariate analysis in Cohort 1, we found that the stroma-immune score was independent of TSR (Fig. 4C).

### Stroma-immune score and intraepithelial-immune score

We found that for both CD3 and CD8, intraepithelial immune cell density was highly correlated with stromal immune cell density in Cohort 1 (Additional file 1: Fig. S7A, B). For stroma-immune score (percentile) and intraepithelial-immune score (percentile), a strong correlation was observed (r = 0.70, P < 0.001; Additional file 1: Fig. S7C). Patients were classified as high, intermediate, and low intraepithelial-immune scores by 37.0% and 64.5% thresholds. Patients with high intraepithelial-immune scores had prolonged OS (HR for high vs. low 0.36, 95% CI 0.22–0.61, P < 0.001) at Cohort 1. We found that the intraepithelial-immune score was not independent of the stroma-immune score in multivariate analysis (Additional file 1: Fig. S7D).

### The added prognostic value of the stroma-immune score

**Fig. 5:**
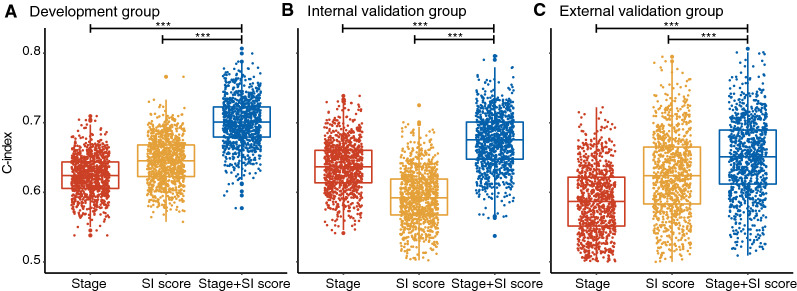
The added prognostic value of stroma-immune score using a 1000× bootstrap strategy (***P < 0.001 after Bonferroni correction). **A** Development group. **B** Internal validation group. **C** External validation group. *SI score* stroma-immune score

We obtained C-index distributions of TNM stage, stroma-immune score, and TNM stage plus stroma-immune score in the development, internal and external validation groups with the bootstrap method. In the development group, the combination of the TNM stage and the stroma-immune score showed better discrimination power than the TNM stage alone (mean C-index: 0.70 vs. 0.62, P < 0.001 after Bonferroni correction; Fig. 5A). The result was confirmed in the internal validation group (0.67 vs. 0.64, P < 0.001 after Bonferroni correction ; Fig. 5B) and external validation group (0.65 vs. 0.58, P < 0.001 after Bonferroni correction; Fig. 5C).

## Discussion

To quantify the immune infiltration within the stroma region in patients with II–III CRC, we designed a deep learning-based pipeline for CD3^+^ and CD8^+^ T-cells calculation on IHC-stained WSIs. A scoring system, named stroma-immune score, was established. Its added prognostic value was evaluated and validated in development, internal, and external validation groups.

The prognosis value of immune infiltrates quantification in CRC has been supported by mounting evidence [5, 7, 18, 19]. Especially, immune infiltration of CD3^+^ and CD8^+^ T-cells subsets in tumor regions has been widely confirmed to be associated with prognosis. CD3 is a common membrane marker for mature T lymphocytes and can be used to represent total T lymphocytes. CD8^+^ cytotoxic T cells are the main antitumor T lymphocyte subsets [20]. Previous studies have shown that remodeling of the extracellular matrix, also known as the stroma, can act as a physical barrier, limiting immune cells' access to cancer cells [21–24]. Increased tumor-stromal composition and decreased intratumoral infiltrating lymphocytes are associated with poor overall survival [24]. A recent study by Reichiling et al. suggests that the prognosis evaluation of CRC acquires further insight into the stromal immune infiltration (CD3^+^ and CD8^+^ T-cells) in addition to intrinsic tumor variables [25]. Moreover, Yoo et al. quantified intraepithelial TILs and stromal TILs separately to describe the landscape of the tumor-immune microenvironment [7]. We developed a deep learning-based stroma-immune score that takes CD3^+^ and CD8^+^ T-cells in the stroma region into account to reveal patient prognosis in CRC. Our study showed that the fully automated quantified stroma-immune score enables prognostic stratification for stage II–III CRC, corroborating the significant role of stromal immune infiltration in the tumor-immune microenvironment. In addition, the stroma-immune score remained a statistically significant predictor for OS when stratified by TNM stage, age, sex, and tumor site, and MSI status, except for the treatment modalities. Despite no significant OS difference was found for the stroma-immune score in both adjuvant chemotherapy and surgery-only groups, we could observe the positive trends. However, further studies are still needed to explore whether the stroma-immune score can help assess patients’ benefit from different treatment modalities. On the other hand, it is noteworthy that our proposed stroma-immune score pipeline could be more easily translated into routine clinical use regarding its reproducibility and reliability compared to subjective evaluation.

Since the TSR has been proven to be an independent prognostic factor for patients with CRC [10, 26], we also investigated the relationship between stroma-immune score and TSR. We found that the stroma-immune score was independent of TSR in multivariate analysis, and patients with stroma-low and high stroma-immune scores had the most favorable survival. In contrast, patients with stroma-high and low stroma-immune scores had the worst survival. These results were consistent with previous studies that patients with stroma-low were associated with a higher survival rate, and patients with high TILs tend to have a better outcome [7, 10, 26]. Additionally, the CD3^+^ and CD8^+^ T-cells within the tumor epithelium region were also analyzed in our study. We observed that the stroma-immune score was strongly correlated with the intraepithelial-immune score. In addition, the stroma-immune score showed superior prognostic value compared with the intraepithelial-immune score. This result supported the idea of focusing on the immune infiltrates in the stroma and also demonstrated that our proposed stroma-immune score may be sufficient for OS prediction in patients with stage II–III CRC.

The tumor microenvironment characteristics have been shown to allow further insight into patients’ prognosis in most solid tumors, including CRC [27, 28]. Unlike the Immunoscore [5], which quantifies CD3^+^ and CD8^+^ T-cells at the tumor core and the invasive margin, our proposed scoring strategy specifically focuses on the stroma region for these two types of immune cells density. As far as we know, Immunoscore takes the tumor region as a whole, ignoring the tumor microenvironment information of specific tissue types, such as immune expression in the stroma region, might be insufficient to capture the biological complexity. Additionally, even in the tumor core region, the stroma also serves an important role in the tumor microenvironment [29, 30]. We also observed that the invasive margin belonged to our automated segmented stroma region (Fig. 1C). Besides, most TILs were located in the stroma rather than tumor epithelium (mean density [cells/mm^2^] of CD3^+^ T-cell: 1304 vs. 355; CD8^+^: 472 vs. 141). Therefore, our study considered only the CD3^+^ and CD8^+^ T-cell in the stroma region to develop the stroma-immune score. As expected, our results showed that the stroma-immune score demonstrated higher discrimination performance for OS prediction compared with the previous study (C-index 0.63 vs. 0.58). However, additional studies should be performed to compare the stroma-immune score with Immunoscore directly.

The study has limitations. First, limited sample sizes were used to evaluate the prognostic value of the stroma-immune score, especially for the external validation group. Second, the presented results still require further prospective and widespread validation. Furthermore, considering the complexity of the tumor immune microenvironment, the prognostic values of other intratumoral-infiltrating T-cell subgroups, such as CD4^+^, FOXP3^+^ T-cells, are needed for further exploring. Moreover, the prognostic value of the spatial distribution of immune invasion in tumor stroma or other tissue types is also one of the research directions we are interested in.

In conclusion, with deep learning, we built a fully automated pipeline to quantify CD3^+^ and CD8^+^ T-cells densities in the stroma region on IHC-stained WSIs of stage II–III CRC. A stroma-immune score was calculated via digital pathology image analysis. We further used two groups to validate the prognostic value of stroma-immune score for OS. The stroma-immune score we proposed could be easily translated into routine pathologic assessment regarding its reproducibility and reliability.

## Supplementary Information

**Additional file 1: Figure S1.** Study profile and IHC tiles dataset generation. **Figure S2–S4.** Kaplan–Meier survival analysis for patients stratified by TNM stage, age, sex, tumor site, MSI status, and treatment modalities. **Figure S5.** Using the pipeline to predict the prognosis of one patient with CD3 and CD8 WSIs. **Figure S6.** Kaplan–Meier survival curve of overall survival of stroma-low vs. stroma-high groups. **Figure S7.** Stroma-immune score vs. intraepithelial-immune score.

## Data Availability

The datasets used and/or analyzed during the current study are available from the corresponding author on reasonable request.
