# Supplementary material for: A deep learning quantified stroma-immune score to predict survival of patients with stage II–III colorectal cancer
Source: Cancer Cell Int. 2021 Oct 30;21:585. doi: 10.1186/s12935-021-02297-w (PMC8557607; doi:10.1186/s12935-021-02297-w)
Supplement: Supplementary file 1 — Additional file 1: Figure S1. Study profile and IHC tiles dataset generation. Figure S2–S4. Kaplan–Meier survival analysis for patients stratified by TNM stage, age, sex, tumor site, MSI status, and treatment modalities. Figure S5. Using the pipeline to predict the prognosis of one patient with CD3 and CD8 WSIs. Figure S6. Kaplan–Meier survival curve of overall survival of stroma-low vs. stroma-high groups. Figure S7. Stroma-immune score vs. intraepithelial-immune score. [file 12935_2021_2297_MOESM1_ESM.pdf]

Additional file for

A deep learning quantified stroma-immune score to predict survival of patients with stage II–III colorectal cancer

Supplementary methods

1. Immunohistochemical staining

The most invasive part of the primary tumor was chosen from the Paraffin tissue block from each patient. Two 4μm sections were cut from the block for immunohistochemistry. Several steps were taken: deparaffinage, antigen retrieval solution (10× concentrate, Novocastra, Leica) and primary (human anti-CD3 [Gene Tech, Cat# GT200229] and anti-CD8 [Cat# GT211229] rabbit monoclonal antibodies) and secondary (rabbit-anti-mouse IgG, Bond Refine Detection Kit, Leica) antibodies, according to the manufacturer's recommendations in an automated Bench Mark Ventana. Finally, sections were subsequently incubated with 3,3-Diaminobenzidine (DAB), counterstained with Haematoxylin, and mounted with special glue. The internal positive control was used for quality assurance.

2. The IHC tiles datasets generation

The stained tissue sections were imaged using digital Whole Slide Scanning (Aperio-AT2, Leica, USA) at 40× magnification. Then, in the development group, contiguous tissue areas were manually annotated and tessellated from 242 CD3 and CD8 slides of 121 patients, creating an IHC tiles training dataset consisting of 154.4k non-overlapping tissue tiles of dimension 224 × 224 $pixel^2$ (0.504 × 0.504 μm^2). An IHC tiles test dataset consisting of 22.5k tissue tiles was also established from 114 slides of 57 patients in the validation group.

Additional figures

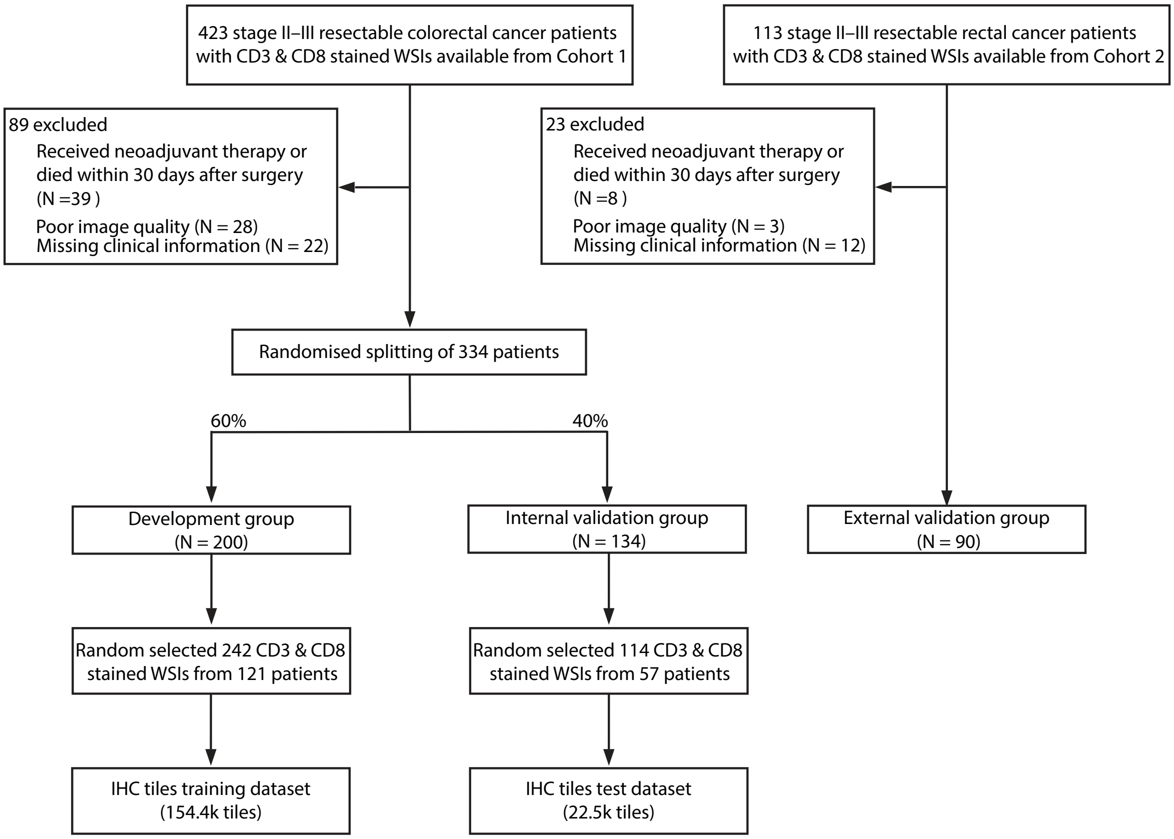

Additional file 1: Fig. S1 Study profile and IHC tiles dataset generation. IHC, immunohistochemical; WSI, whole-slide image; Cohort 1, Guangdong Provincial People's Hospital; Cohort 2, the Sixth Affiliated Hospital of Sun Yat-sen University.

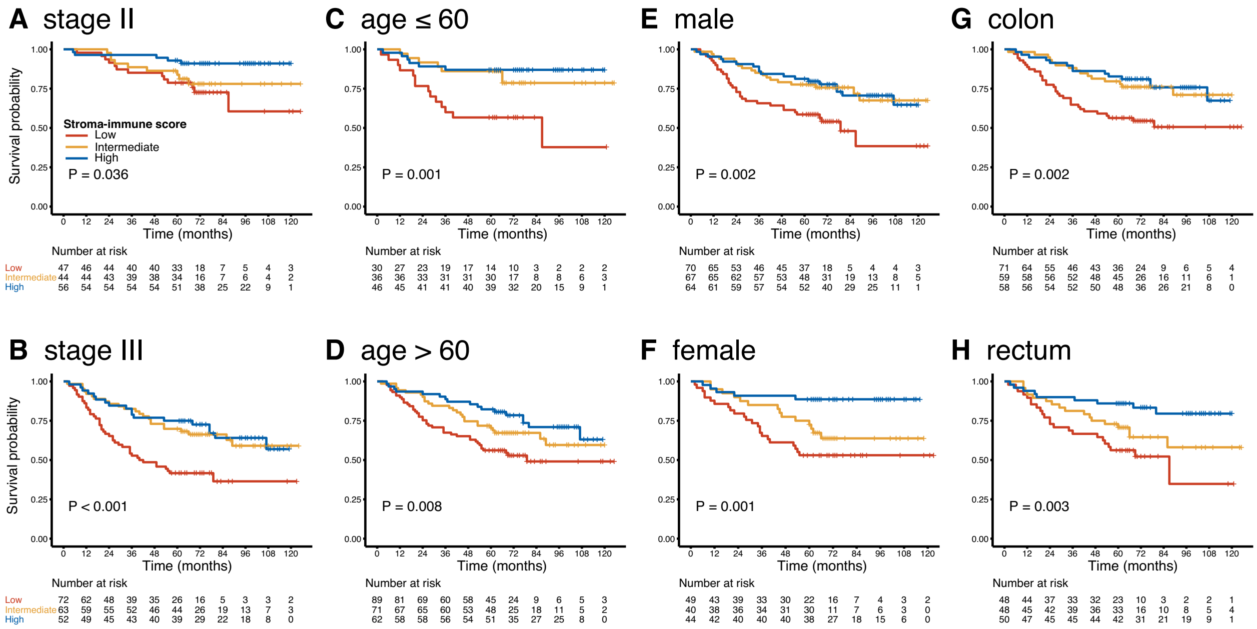

Additional file 1: Fig. S2 Kaplan–Meier survival analysis for patients stratified by TNM stage, age, sex, and tumor site. TNM, tumor-node-metastasis.

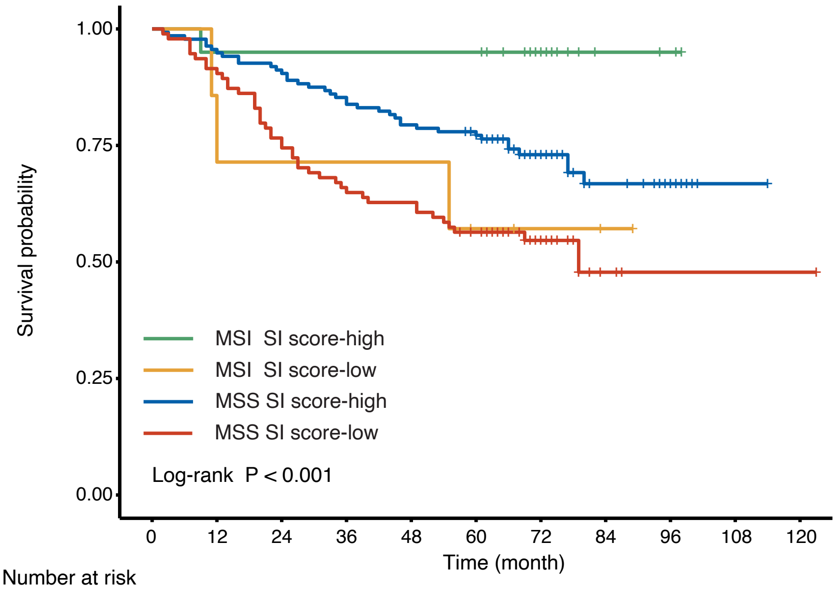

Number at risk

MSI SI score-high	20	19	19	19	19	19	13	5	3	0	0
MSI SI score-low	7	6	5	5	5	3	2	1	0	0	0
MSS SI score-high	136	130	124	116	108	101	51	25	15	1	0
MSS SI score-low	94	86	72	62	59	46	23	3	1	1	1

Additional file 1: Fig. S3 Kaplan–Meier survival analysis of overall survival stratified by MSI status. MSI, microsatellite instability; MSS, microsatellite stable; SI score, stroma-immune score.

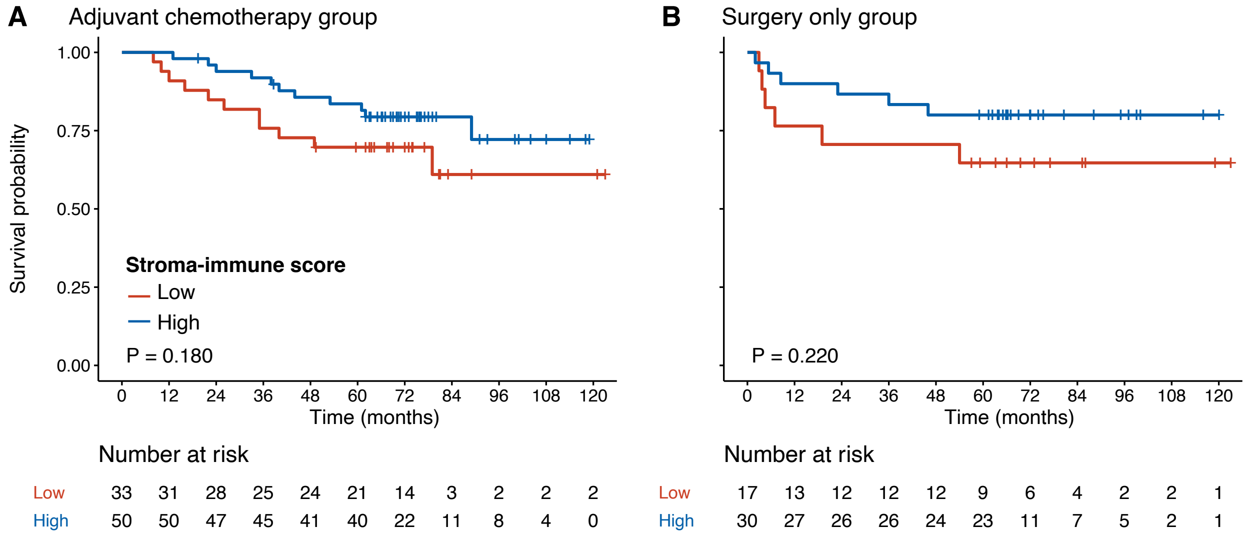

Additional file 1: Fig. S4 Kaplan–Meier survival analysis of overall survival stratified by treatment modality.

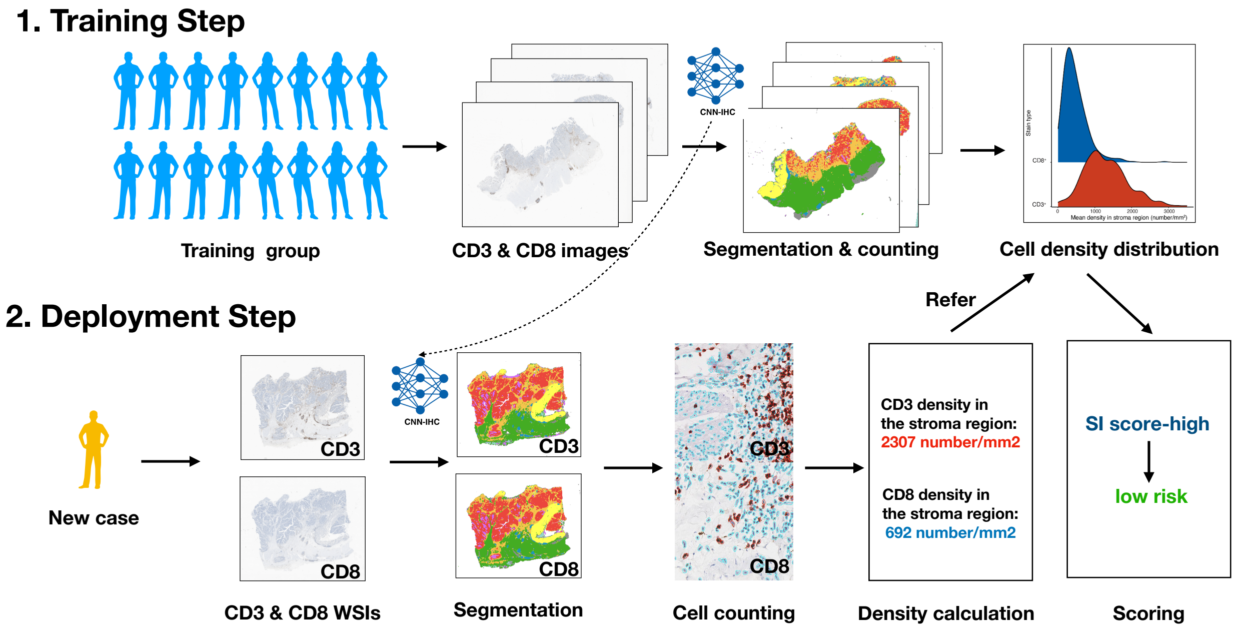

Additional file 1: Fig. S5 Using the pipeline to predict the prognosis of one patient with CD3 and CD8 WSIs. WSI, whole-slide image; SI score, stroma-immune score.

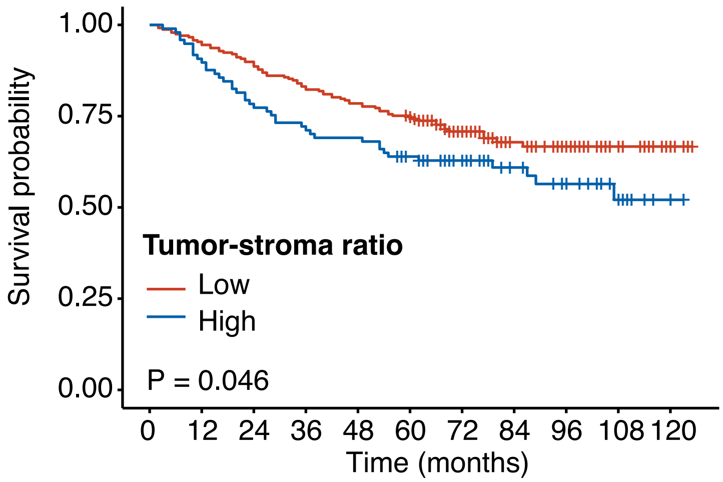

Number at risk

Stroma-low	237	226	213	197	186	168	98	57	44	23	8
Stroma-high	97	88	76	70	67	59	45	28	23	12	3

Additional file 1: Fig. S6 Kaplan–Meier survival curve of overall survival of stroma-low vs. stroma-high groups.

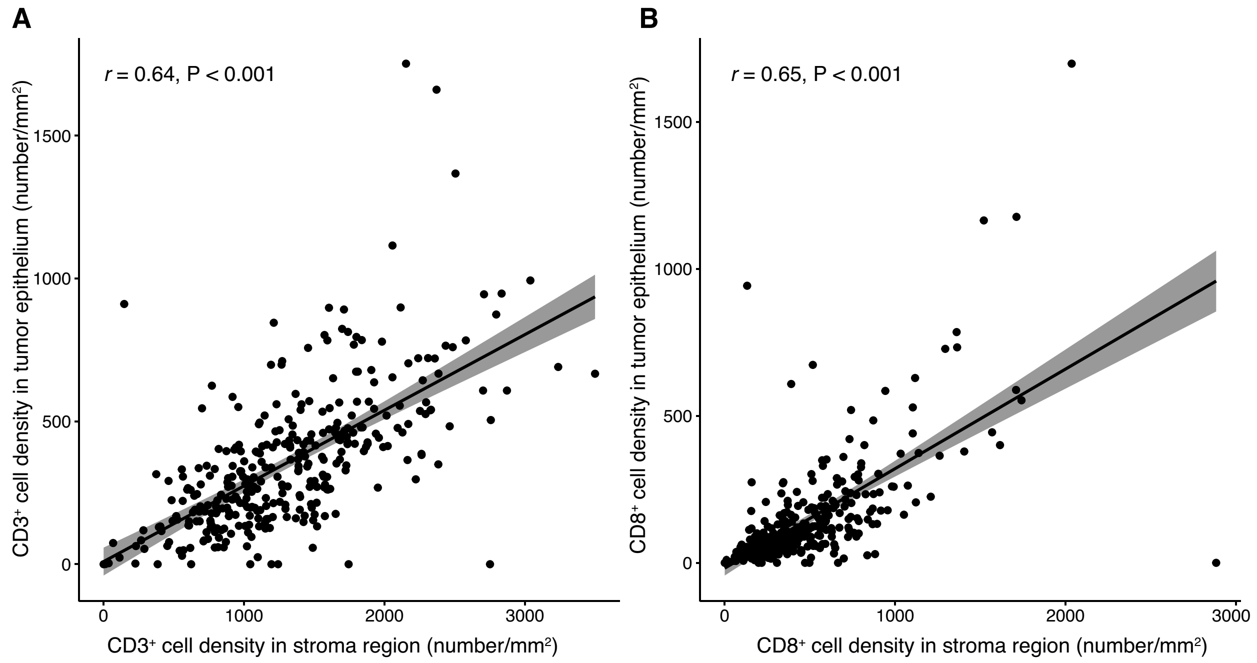

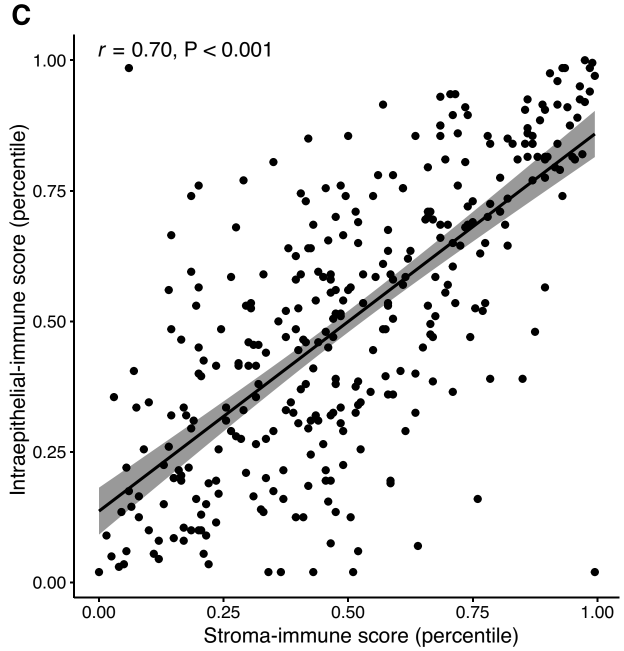

D

Characteristic	HR	95% CI	P
**Intraepithelial-immune score**			
Low	1.00		
Intermediate	1.19	0.77-1.84	0.429
High	0.57	0.29-1.10	0.095
**Stroma-immune score**			
Low	1.00		
Intermediate	0.54	0.34-0.85	0.008
High	0.49	0.26-0.91	0.025

Additional file 1: Fig. S7 Stroma-immune score vs. intraepithelial-immune score. (A) Correlation of $CD3^+$ cell density in stroma region and tumor epithelium. (B) Correlation of $CD8^+$ cell density in stroma region and tumor epithelium. (C) Correlation of stroma-immune score and intraepithelial-immune score in percentile. (D) Multivariate analysis of stroma-immune score and intraepithelial-immune score. HR, hazard ratio; CI, confidence interval.
